# A novel anastomotic vacuum-assisted closure system for cervical anastomotic fistula after three-incisional esophagectomy

**DOI:** 10.1093/gastro/goad023

**Published:** 2023-06-07

**Authors:** Yu-Zhen Zheng, Shen-Shen Fu, Xian-Yu Qin, Xing-Ping Yang, Hao-Sheng Zheng, Zui Liu, Shi-Yun He, Wei-Jie Cai, Jian Tan, Bo-Zhu Jian, Hong-Ying Liao

**Affiliations:** Department of Thoracic Surgery, The Sixth Affiliated Hospital, Sun Yat-sen University, Guangzhou, Guangdong, P. R. China; Department of Esophageal Surgery, The Sixth Affiliated Hospital, Sun Yat-sen University, Guangzhou, Guangdong, P. R. China; Department of Ultrasonography, Guangzhou First People’s Hospital, School of Medicine, South China University of Technology, Guangzhou, Guangdong, P. R. China; Department of Thoracic Surgery, The Sixth Affiliated Hospital, Sun Yat-sen University, Guangzhou, Guangdong, P. R. China; Department of Esophageal Surgery, The Sixth Affiliated Hospital, Sun Yat-sen University, Guangzhou, Guangdong, P. R. China; Department of Thoracic Surgery, The Sixth Affiliated Hospital, Sun Yat-sen University, Guangzhou, Guangdong, P. R. China; Department of Esophageal Surgery, The Sixth Affiliated Hospital, Sun Yat-sen University, Guangzhou, Guangdong, P. R. China; Department of Thoracic Surgery, The Sixth Affiliated Hospital, Sun Yat-sen University, Guangzhou, Guangdong, P. R. China; Department of Esophageal Surgery, The Sixth Affiliated Hospital, Sun Yat-sen University, Guangzhou, Guangdong, P. R. China; Department of Thoracic Surgery, The Sixth Affiliated Hospital, Sun Yat-sen University, Guangzhou, Guangdong, P. R. China; Department of Esophageal Surgery, The Sixth Affiliated Hospital, Sun Yat-sen University, Guangzhou, Guangdong, P. R. China; Department of Thoracic Surgery, The Sixth Affiliated Hospital, Sun Yat-sen University, Guangzhou, Guangdong, P. R. China; Department of Esophageal Surgery, The Sixth Affiliated Hospital, Sun Yat-sen University, Guangzhou, Guangdong, P. R. China; Department of Thoracic Surgery, The Sixth Affiliated Hospital, Sun Yat-sen University, Guangzhou, Guangdong, P. R. China; Department of Esophageal Surgery, The Sixth Affiliated Hospital, Sun Yat-sen University, Guangzhou, Guangdong, P. R. China; Department of Thoracic Surgery, The Sixth Affiliated Hospital, Sun Yat-sen University, Guangzhou, Guangdong, P. R. China; Department of Esophageal Surgery, The Sixth Affiliated Hospital, Sun Yat-sen University, Guangzhou, Guangdong, P. R. China; Department of Thoracic Surgery, The Sixth Affiliated Hospital, Sun Yat-sen University, Guangzhou, Guangdong, P. R. China; Department of Esophageal Surgery, The Sixth Affiliated Hospital, Sun Yat-sen University, Guangzhou, Guangdong, P. R. China; Department of Thoracic Surgery, The Sixth Affiliated Hospital, Sun Yat-sen University, Guangzhou, Guangdong, P. R. China; Department of Esophageal Surgery, The Sixth Affiliated Hospital, Sun Yat-sen University, Guangzhou, Guangdong, P. R. China

## Introduction

Surgery remains the mainstay treatment for localized esophageal squamous cell carcinoma (ESCC). In recent years, three-incision McKeown esophagectomy has been widely applied for operable ESCC considering its superiority in acquiring extensive lymphadenectomy and long-term survival [1]. Cervical anastomotic fistula is one of the most dreaded complications in patients undergoing three-incision esophagectomy; it occurs in almost 10%–25% of patients and is responsible for almost 40% of post-operative deaths [2, 3]. Most surgeons prefer conservative approaches including perianastomotic drainage, total parenteral nutrition, nasogastric decompression, and the use of broad-spectrum antibiotics for cervical anastomotic fistula [4, 5]. However, the recovery period under this method is commonly long and unsatisfactory. Until now, the optimal treatment for cervical anastomotic fistula has not yet been established. In this study, we describe a flushing-drainage system under negative pressure conditions to treat cervical anastomotic fistula, named anastomotic vacuum-assisted closure (AVAC).

## Procedure of AVAC

**Figure 1.**
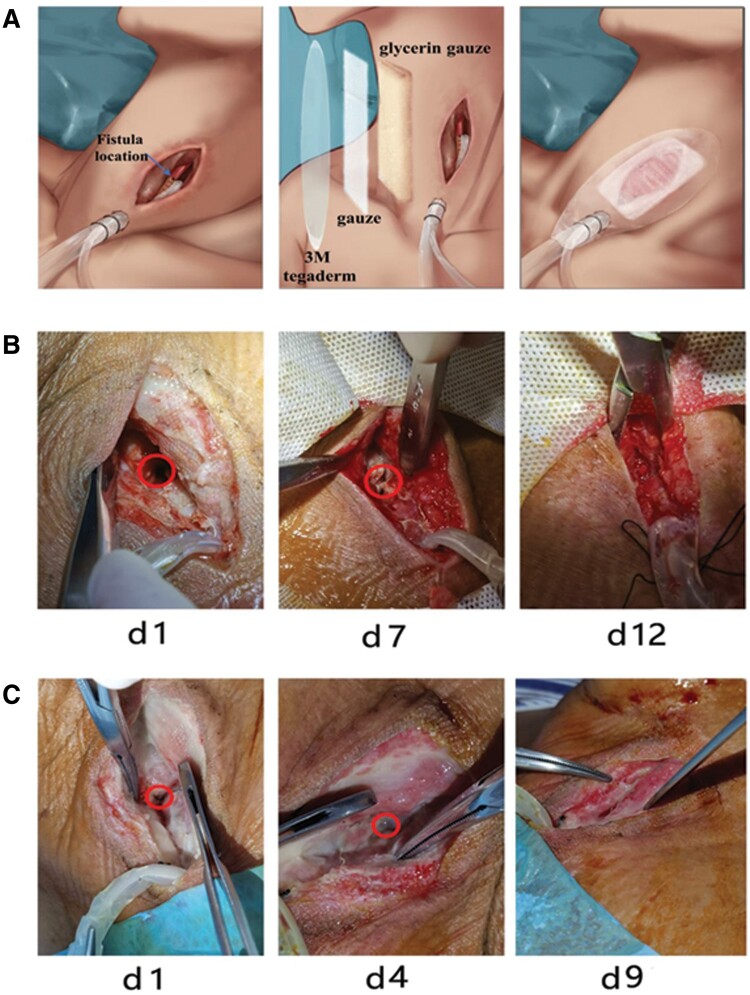
Design of the anastomotic vacuum-assisted closure (AVAC) system and application in the clinic. (A) Procedure of AVAC for cervical anastomotic fistula; (B) a patient was diagnosed with anastomotic fistula on the post-operative 8th day and the leakage was cured with AVAC therapy in 12 days; (C) a patient was diagnosed with anastomotic fistula on the post-operative 9th day and the leakage was cured with AVAC therapy in 9 days.

After diagnosis of an anastomotic fistula, we would first predict the leakage location based on the esophagogram and computed tomography (CT) scan and then open the cervical wound. The tissue space around sternocleidomastoid should be dissociated for sufficient exposure of the esophagus. Patients would be asked to swallow methylthionine and cough, and the site with blue saliva or bubble outflow is the leakage location. For those without significant saliva or bubble outflow, methylthionine swallowing test would be performed. Then, a drainage tube with internal notches and flushing arrangement was placed through a subcutaneous tunnel. The head of the drainage tube was placed close to the leakage. The interval notch is indispensable to guarantee an adequate drainage effect under negative pressure conditions. Then, glycerin gauze packing was applied to generate a sealed environment. Finally, a 3M Tegaderm was applied to cover the wound. Normal saline was used to flush the wound with the negative pressure as 0.04 MPa and the speed is 10–40 mL/h, depending on the secretion amount. Then, a persistent negative pressure suction would be provided to the environment to ensure effective drainage (Figure 1A).

To assess the degree of leakage recovery, we defined two milestone tests to manifest the degree of leakage recovery. The first is the swallowing and coughing test. According to the test, the patient would be asked to swallow and cough under the exposure of the wound. No observable saliva or bubble indicated the first step of leakage healing. The second is the drinking test. In this test, the patient would be asked to drink little water under the exposure of the wound. No observable exudation indicated the phased completion of fistula recovery. Then, we would gently pull out the drainage tube almost 0.5 cm each time and modify the flushing speed to almost 5 mL/h. In this condition, the high negative pressure would accelerate tissue round-up and healing. In this process, migratory mucosa should be carefully recognized and excised to avoid the development of a sinus tract, which is a common failure pattern in treating anastomotic leakage. Finally, the superficial wound would be sutured.

## Case presentation

Five patients who underwent McKeown esophagectomy and encountered post-operative cervical anastomotic fistula were selected for this novel approach. Cervical anastomotic fistulas were diagnosed between Days 5 and 10 post-operatively. The diagnosis of cervical anastomotic fistula was made by the comprehensive assessment of esophagogram, CT scan, and clinical symptoms. The median time duration to pass the first test, second test, and final sewing was 4, 6, and 13 days. Finally, all leakages were successfully managed (Figure 1B and C).

## Discussion

Cervical anastomotic fistula is a common morbidity after three-incisional esophagectomy. Traditional therapy for cervical anastomotic fistula requires daily dressing changes. However, the leakage heals slowly and the patient is in pain. In addition, when the fistula is not satisfactorily controlled, terrible co-morbidities such as mediastinitis may develop [6].

In this study, we describe a novel system for this common post-operative complication that to our knowledge has not yet been reported. This system is modified from the vacuum drainage system that has been applied in the infection of a superficial wound [7, 8]. Unlike general wound infections, cervical wound infection originates from anastomotic leakage. Therefore, this vacuum drainage system is designed to accelerate the healing process of the leakage rather than the entire wound. With this purpose, the drainage tube is designed with internal notches to guarantee adequate drainage even under high negative pressure. Besides, we insert a flushing tube inside the drainage tube; in the proposed system, the flushing tube and the drainage tube are placed around the leakage. In addition, the flushing rate is set at 40 mL/h because this velocity is able to take away secretions and keep the tissue moist, which is critical in helping to promote tissue healing. We hypothesize that, if the secreta from the leakage is adequately drained, the infection of the cervical wound will disappear. Therefore, in the proposed system, instead of draining the entire wound, we packed it with glycerin gauze and covered it with 3M Tegaderm. This design concentrates the negative pressure on the deep and narrow space around the leakage, which makes this system different from the traditional vacuum drainage system for superficial wound infections.

In our opinion, our proposed system makes the healing process work from the inside out, thus avoiding a common cause of failure, namely the completion of wound healing before leakage healing. We proposed two milestone tests to manifest the physiological process of fistula healing, which highlights significant clinical implications. Notably, even if patients pass the second test, the healing mucosa is fragile and minimal leakage may exist, so we proposed two suggestions to improve the success rate. The first suggestion is to avoid immediate oral feeding and incision sewing. The second suggestion is to avoid immediately removing the drainage tube but to pull it out slowly in almost 2 or 3 days. According to our method, the dressing could be changed every 2–3 days, which would save doctors’ time and relieve patients’ pain. It is plausible that the proposed system is useful, since all recruited patients with cervical leakage under this method healed within a short time.

There are some limitations for this device. First, the device is most efficient for patients with anterior wall leakage, but less efficient when the leakage is located in the posterior wall and invalid for patients with leakage to the chest cavity. Second, because the device contains some gauze and a vacuum drainage, the packing procedure and the vacuum pressure make it painful for the patients.

In conclusion, the proposed vacuum drainage system appears to be suitable for cervical anastomotic fistula. This model optimizes the technique procedure based on easily collected material and highlights significant clinical implications.
